# Integration scheme of nanoscale resistive switching memory using bottom-up processes at room temperature for high-density memory applications

**DOI:** 10.1038/srep28966

**Published:** 2016-07-01

**Authors:** Un-Bin Han, Jang-Sik Lee

**Affiliations:** 1 Department of Materials Science and Engineering, Pohang University of Science and Technology (POSTECH), Pohang 790-784, Korea

## Abstract

A facile and versatile scheme is demonstrated to fabricate nanoscale resistive switching memory devices that exhibit reliable bipolar switching behavior. A solution process is used to synthesize the copper oxide layer into 250-nm via-holes that had been patterned in Si wafers. Direct bottom-up filling of copper oxide can facilitate fabrication of nanoscale memory devices without using vacuum deposition and etching processes. In addition, all materials and processes are CMOS compatible, and especially, the devices can be fabricated at room temperature. Nanoscale memory devices synthesized on wafers having 250-nm via-holes showed reproducible resistive switching programmable memory characteristics with reasonable endurance and data retention properties. This integration strategy provides a solution to overcome the scaling limit of current memory device fabrication methods.

Resistive random access memory (ReRAM) has been investigated to be the most important candidate for next-generation memory devices in terms of switching speed, power consumption, and scalability^1^,^2^,^3^,^4^,^5^,^6^,^7^,^8^,^9^,^10^. In ReRAM, transition metal oxides are commonly used as resistive switching layers^2^,^8^,^11^,^12^,^13^. Synthesis of metal oxide layers is one of the most important factors in development of ReRAM. Transition metal oxide layers have been synthesized by vacuum deposition systems with annealing processes^6^,^14^,^15^,^16^.

Because metal oxide-based ReRAMs have many potential advantages, including reliable operations, CMOS compatiability, low power consumption, and scalability, efforts have been devoted to improve the properties of ReRAM by using various transition metal oxides such as Cu_2_O^17^,^18^, NiO^19^,^20^,^21^, TiO_2_^22^,^23^,^24^, and Ta_2_O_5_^25^,^26^. In particular, various approaches have been investigated to improve their fabrication methods with materials design including electrodes^27^,^28^,^29^, resistive switching layers^12^,^30^,^31^,^32^,^33^,^34^, and interfacial layers^35^,^36^,^37^,^38^. However, conventional processes mostly use vacuum and annealing processes to deposit transition metal oxides; these methods require long process time and high-temperature annealing.

In addition, fabrication of high-density nanoscale ReRAM has been an important challenge. High-density memory can be achieved by scaling down of memory cell sizes and 3D stacking with cross-point arrays. However, these methods need accurate and expensive fabrication processes, as well as appropriate materials selection for the vertical stacking process. To solve these problems, an alternative approach is to use electrochemical deposition (ECD) to fabricate thin films. ECD has been used to fabricate metal/insulator/metal (MIM) ReRAM structure^39^,^40^. Au/NiO/Au nanowires with nanoscale diameters were synthesized by ECD into nano-templates and thermal oxidation^41^. In addition, a Cu_2_O layer was reported to be formed by thermal oxidation of a copper film grown on a Si substrate^17^,^18^. In these methods, the metal oxide film is formed by thermal oxidation of the deposited metal layer by post annealing processes. This fabrication method requires additional process steps including thermal oxidation of electrodeposited metals. Considering the thermal budget device fabrication at low temperature is required.

In this paper, we demonstrate ECD Cu_2_O-based ReRAMs with high reproducibility and reliability. The fabrication cost and process time for ReRAM devices can be effectively reduced since the critical step is a solution process and most device fabrication can be done at room temperature (RT) without any additional oxidation/annealing processes. ECD enables the fabrication of integrated memory devices with accurate control of materials during the deposition process and various structures/device functions without encountering a scaling limit. This method can form thin oxide films at atmospheric pressure without vacuum conditions. ECD controls the adsorption of atoms by applying bias to the substrate, and can be used to synthesize metal oxides with accurate thickness and stoichiometry at the nanoscale.

## Experimental

### Synthesis of 250-nm via-hole patterns on silicon wafers

To fabricate nanoscale memory devices, nanoscale via-hole patterns (diameter of 250 nm; thickness of 100 nm) were fabricated on silicon wafers. First plasma-enhanced chemical vapor deposition was used to deposit a 100-nm-thick SiO_2_ layer on the Pt bottom electrode/Ti/SiO_2_/Si substrate. Via-hole patterns (diameter 250 nm) were formed using the optical lithography and etching proesses (KrF lithography and reactive-ion etching)^42^. The SiO_2_ layer was used as the sidewall for device isolation; the Cu_2_O and top electrode of the memory devices were deposited using ECD and E-beam evaporation.

### Fabrication of 250-nm memory devices

We fabricated Cu_2_O-based nanoscale memory devices using 250-nm via-hole structures on silicon wafers. The Cu_2_O as the resistive switching layer of nanoscale memory devices was deposited using ECD. The Cu_2_O layer was synthesized using 0.6 M CuSO_4_·5H_2_O aqueous solution amended with 3 M lactic acid (Sigma Aldrich) to stabilize Cu (II) ions. The aqueous solution was adjusted to pH of 9 by adding 2 M NaOH (Sigma Aldrich) then stirred overnight using a magnetic stirrer. ECD was performed using a potentiostat/galvanostat (Reference 3000, GAMRY) with a three-electrode system. Ag/AgCl (3 M KCl) was used as the reference electrode and carbon was used as the counter electrode. The Cu_2_O layer was deposited at *J* = 1 mA/cm^2^ at 25 °C. The deposition thickness of Cu_2_O was ~70 nm. A Pt layer with 100-nm thickness as the top electrode was deposited using an e-beam evaporator.

### Characterization

A potentiostat/galvanostat was used for ECD and cyclic voltammetry. ECD of Cu_2_O was conducted using galvanostatic polarization. A cyclic voltammogram was measured using potential scanning from open circuit potential (OCP, vs. Ag/AgCl). The surface morphology and composition of Cu_2_O-based nanoscale memory device were observed using a high-resolution transmission electron microscope (HR-(S)TEM-I; JEM 2100F with a Cs corrector on STEM, JEOL). Before TEM observations, the samples were prepared using a focused ion beam (FIB; Helios, FEI). The crystal structure and phase of the Cu_2_O thin films were investigated using x-ray diffraction (XRD, D/MAX-2500/PC, RIGAKU) using Cu Kα radiation (λ = 1.54178 Å). The electrical properties of nanoscale memory devices were measured using a semiconductor parameter analyzer (SPA, 4200-SCS, KEITHLEY). All electrical measurements were performed at RT and atmospheric condition, except data retention property measured at 85 °C.

## Results and Discussion

**Figure 1:**
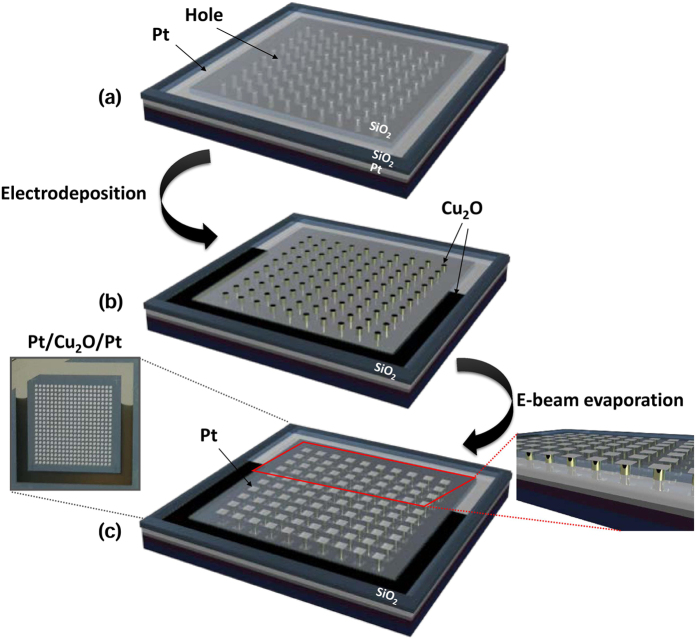
Experimental procedures for fabrication of Cu_2_O-based nanoscale memory devices with 250-nm via-holes patterned on wafer. (**a**) 250-nm via-hole structures fabricated by lithography and etching processes, (**b**) Cu_2_O layer formed by ECD, (**c**) top electrode (Pt) deposition by E-beam evaporation (Left image: optical image of the fabricated sample, Right image: cross-sectional schematic view of the memory device).

The 250-nm sized Cu_2_O-based ReRAMs with solution processes were fabricated as follows (Fig. 1). A Si wafer having an array of 250-nm via holes was fabricated using lithography (Fig. 1a). A Pt layer was used as the bottom electrode as well as a seed layer for ECD. The Cu_2_O film as the resistive layer was deposited using ECD (Fig. 1b). ECD induces an electric bias in a solution containing the precursor of the material, and this bias causes an oxidation/reduction reaction which precipitates ions to form the thin film (Figure S1). The Cu_2_O layer synthesized with ECD on a Pt via-hole substrate can be used as the resistive switching layer at RT without annealing processes. A Cu_2_O layer was grown in Pt via-hole of 250 nm diameter by applying bias to the Pt bottom electrode. Finally, e-beam evaporation was used to deposit the top Pt electrode (~100 nm) on the Cu_2_O layer using a shadow mask (Fig. 1c). We chose a symmetric cell with Pt electrodes to avoid unintended factors that might be caused when top and/or bottom electrodes were constructed with reactive metals.

**Figure 2:**
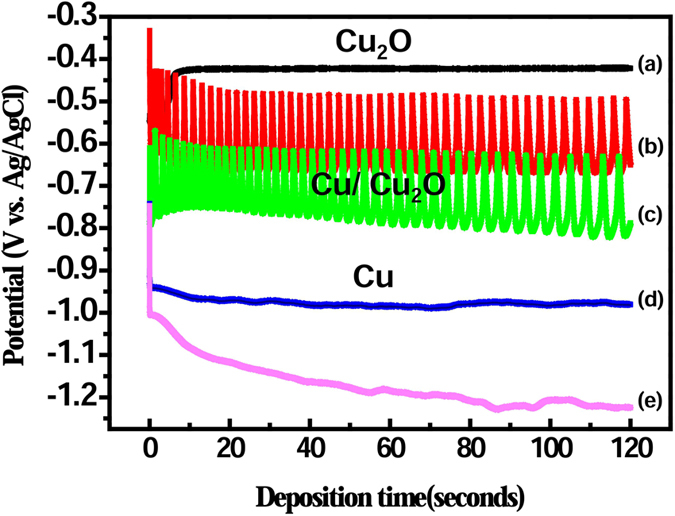
Potential profiles during ECD performed at room temperature and pH of 9 at various applied current densities recorded for 120 s: (**a**) 1 mA/cm^2^, (**b**) 5 mA/cm^2^, (**c**) 10 mA/cm^2^, (**d**) 15 mA/cm^2^, and (**e**) 20 mA/cm^2^.

Potential oscillation was observed during ECD (Fig. 2). Potential vs. deposition time was measured at pH 9 and *J* = 1, 5, 10, 15, and 20 A/cm^2^ for 120 s. At *J* = 1 mA/cm^2^ and negative potential ≤−0.45 V, pure Cu_2_O films were obtained (Fig. 2a). In contrast, at *J* ≥ 15 mA/cm^2^ and negative potential ≥−0.95 V, pure Cu films were obtained (Fig. 2d,e). At *J* = 5 and 10 mA/cm^2^, Cu-Cu_2_O composite films were obtained, and the range of potential oscillated by about 0.2 V (Fig. 2b,c). Previous research reported a similar trend during ECD^43^,^44^. The oscillations in potential are induced by the formation and breakdown of a Cu_2_O at the interface between electrode and solution, and by alteration of the surface pH during ECD^43^. During the growth process, the electrode potential of Cu_2_O at equilibrium is higher than that of Cu. Therefore, a Cu_2_O layer can be deposited on the electrode. The pH in the solution decreased locally to deplete OH^−^ and Cu^2+^ in the migration region. OH^−^ depletion causes the standard electrode potential (*E*^o^) of Cu_2_O to become lower than *E*^o^ of the reaction. The decrease of the pH results in the formation of metallic Cu. However, when the nucleation and growth of Cu occur, the quantity of Cu^2+^ ions in the migration region gradually decrease; eventually, the concentration of OH^−^ ion increases again, and *E*^o^ of Cu_2_O exceeds *E*^o^ of the reaction. Therefore, potential oscillations occur due to formation of Cu-Cu_2_O composite films formed during ECD.

Accordingly, pure Cu_2_O films were obtained at low *J*. In addition, Cu_2_O films are thermodynamically favored in alkaline solution due to formation of Cu_2_O by combination of OH^−^ ions with Cu^2+^ ions in the solution. Consequently, Cu_2_O for the resistive switching layer of nanoscale memory devices was synthesized at *J* = 1 mA/cm^2^ and pH of 9.

**Figure 3:**
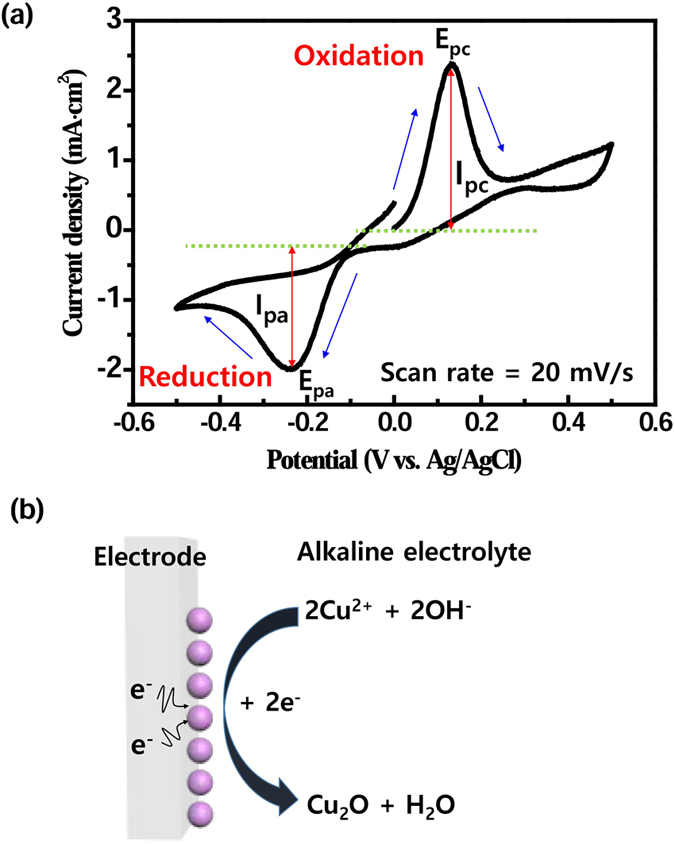
Cyclic voltammogram of Cu_2_O on electrode recorded in 0.6 M CuSO_4_ solution at potential sweep rate of 20 mV/s. (**a**) Reduction peak of Cu^2+^ to Cu_2_O at −0.23 V vs. Ag/AgCl (I_pa_: peak anodic current; I_pc_: peak cathodic current; E_pa_: oxidation peak; E_pc_: reduction peak). (**b**) Mechanism for reduction of Cu^2+^ and OH^−^ ions in solution.

To investigate the electrochemical properties and mechanism for redox reaction of Cu_2_O, the electrical behavior of Cu ions in the CuSO_4_ solution was observed using a potentiostat/galvanostat. The cyclic voltammogram of Cu_2_O was obtained during ECD at a scan rate of 20 mV/s (Fig. 3). Oxidation and reduction showed peak cathodic *i*_pc_ and anodic *i*_pa_ currents (Fig. 3a). The cathodic and anodic peaks showed a potential range between −0.5 V and 0.5 V. The oxidation *E*_pa_ and reduction *E*_pc_ peaks were observed at 0.14 V and −0.23 V, respectively. According to the Nernst equation, the electrode potential of Cu and/or Cu_2_O is determined by the Cu^2+^ concentration. During reduction, Cu_2_O film is formed on the electrode surface^45^. The electric potential difference between working electrode and counter electrode generates electrons (e^−^), which combine in solution with Cu^2+^ ions and OH^−^ ions. Then the Cu^2+^ ions are precipitated as Cu_2_O (Fig. 3b):


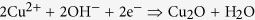


A Cu_2_O film is formed on the electrode by nucleation and growth. An optimized Cu_2_O resistive switching layer was obtained by changing *J* at RT. The oxide layer was formed inside via-holes selectively by using this method.

To confirm crystal structure and composition of thin films using ECD, the XRD analysis of Cu_2_O layer was done (Figure S2). The diffraction peak of the Cu_2_O was obtained using samples deposited at *J* = 1 mA/cm^2^ and RT; results indicate that Cu_2_O crystalline phase is formed on the Pt electrode. The Cu_2_O film showed mostly (111) orientation, mostly due to the (111) oriented Pt bottom electrodes. Additionally, XRD pattern showed no other structures such as metallic Cu or CuO.

**Figure 4:**
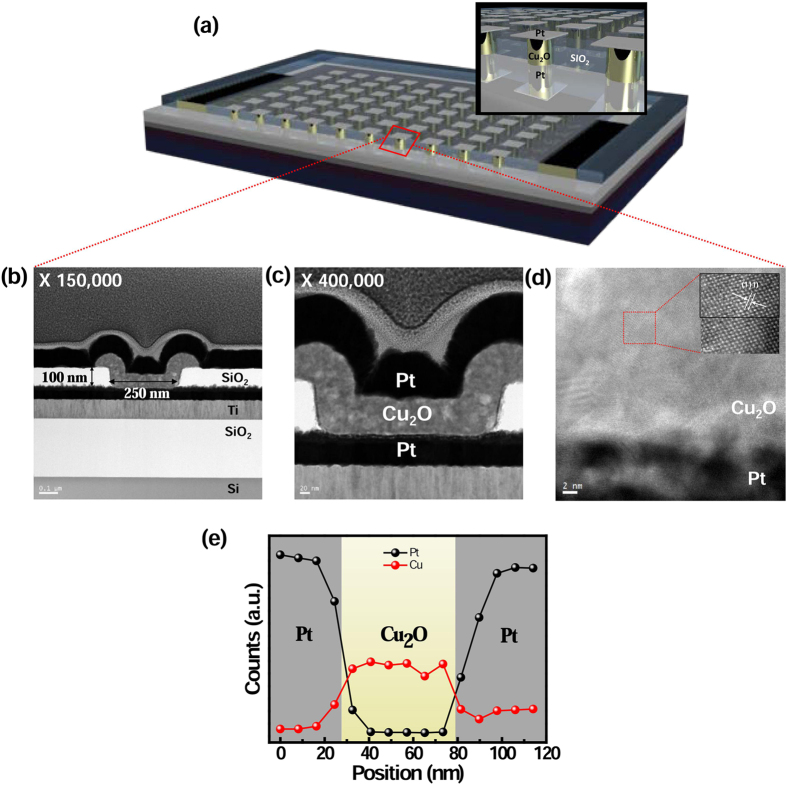
(**a**) Schematic illustration of Pt/Cu_2_O/Pt resistive memory devices. (**b–d**) Cross-sectional TEM images of Pt/Cu_2_O/Pt device at different magnifications, (**e**) EDS composition profile of Pt/Cu_2_O/Pt structure.

Using the aforementioned ECD process, Cu_2_O-based nanoscale memory devices were fabricated in Pt-via hole (diameter of 250 nm) wafer (Fig. 4a). A high-resolution transmission electron microscopy (HR-TEM) image of the Cu_2_O-based ReRAM confirms the 250-nm diameter of the via-hole with the synthesized ECD-based Cu_2_O layer of 70 nm thickness (Fig. 4b–d). Furthermore, EDS composition analysis confirmed the well synthesis of each layer in ReRAM with the Pt-Cu_2_O-Pt structure (Fig. 4e). These results confirm that the bottom-up approach using ECD can successfully be employed to fabricate nanoscale memory devices without vacuum deposition systems and high temperature processes. It is demonstrated that the synthesis of oxide layers with good crystallinity, pure phase, and a smooth interface using ECD.

**Figure 5:**
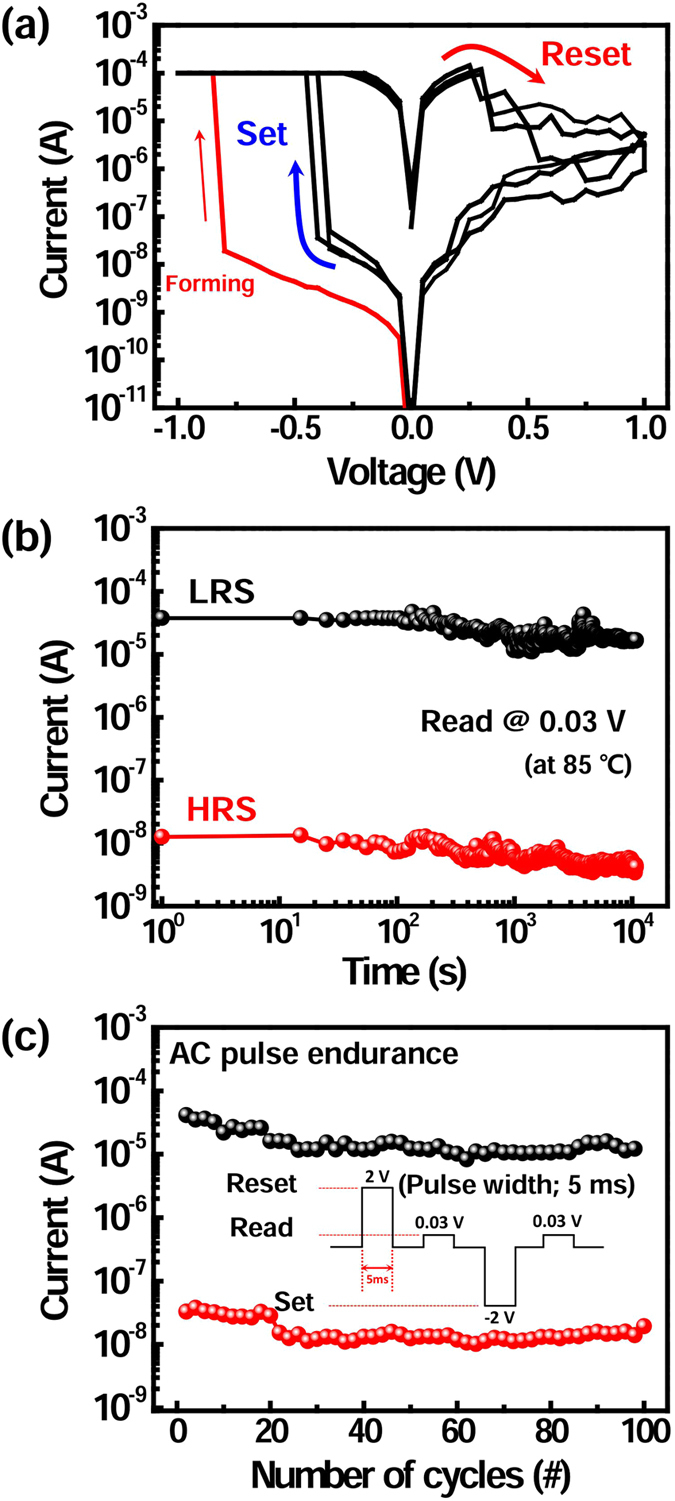
Electrical characteristics of the Pt/Cu_2_O/Pt device with a 250-nm device size. (**a**) I-V curves of Cu_2_O-based nanoscale ReRAM. (**b**) Data retention characteristic of the device. (**c**) AC pulse endurance characteristic of the device (Inset: applied pulse conditions).

The electrical properties of the Cu_2_O-based ReRAMs are shown in Fig. 5. The bottom Pt electrode was grounded; the top Pt electrode was biased. To avoid permanent breakdown, compliance current of 10^−4^ A was set. When negative bias is applied to the top electrode, the conductive filament is formed because of the positively charged Cu ions in CuO_x_. The initial resistance state of the device is changed from high resistance state (HRS) to the low resistance state (LRS), which is called forming operation. The forming process can be done at less than −1 V, which can be attributed to easy diffusion of ionized Cu in the Cu_2_O layer^46^. In addition, the migration of Cu^+^ ions is controlled by applied voltage and bias polarity. Since the forming process provides strong one-directional electric field for a device, it can induce the asymmetric Cu^+^ ion distribution between the bottom and top electrodes, resulting in asymmetric electrical characteristics of the Cu_2_O-based ReRAM. Accordingly, the electrical polarity strongly depends on the initial forming operation^4^. To change the resistance state opposite bias was applied.

After the first forming operation, the conductive filament of the Cu_2_O switching layer is formed by a set process at negative bias. By contrast, when positive bias is applied to the top electrode, the conductive filament is dissolved by a reset process. That is, set voltage of the device is occured at −0.36 V, whereas reset voltage is occured at +0.31 V (Fig. 5a). To examine the reliability of the ReRAM, the time-dependent data retention and program/erase endurance were measured. The retention property was measured at 85 °C. Once the filament was formed in the Cu2O layer, continuous read voltage of 0.03 V was applied to confirm LRS. In addition, HRS retention was measured by applying continuous read voltage of 0.03 V after reset. The on/off ratio was >10^3^, and did not degrade noticeably more than ~10^4^ s (Fig. 5b). Program/erase endurance of the device was evaluated as a function of the number of AC set/reset pulses (Fig. 5c). The inset of Fig. 5c describes set and reset pulse conditions for endurance assessment. After applying set and reset bias pulses, read voltage was followed to read memory states of the ReRAM. We could not observe any noticeable degradation for more than 100 cycles. In addition, cell-to-cell characteristics were measured to confirm the uniformity of memory devices (10 devices, Figure S3). The cumulative probability of HRS and LRS currents are shown in Figure S4. In this paper, we have mainly focused on evaluation of the feasibility of using ECD to fabricate high-density memory devices. Cu_2_O-based ReRAM exhibited reasonable data retention and endurance with uniform electrical properties, which are suitable for non-volatile memory applications.

It is very important to investigate the switching mechanism in RRAM devices. Both cations and anions can participate in switching behaviors. It is also reported that different conditions could result in different predominant defects^47^. Recent studies showed that both cations and anions could be mobile in transition metal oxides^48^. Further work will be carried out to investigate the exact switching mechanism in our memory devices based on defect chemistry.

## Conclusions

In conclusion, we demonstrated ECD to fabricate Cu_2_O-based nanoscale ReRAMs at RT without annealing process. The Cu_2_O layer used as the resistive switching layer in ReRAM was successfully synthesized using bottom-up growth into 250-nm diameter via-holes patterned in Si wafer. The ReRAM exhibited high on/off ratio and reasonable data retention/endurance reliability; all of these traits make it applicable for non-volatile memory applications. This study suggests that ECD may be a simple and cost-effective approach to fabricate next-generation high density memory devices beyond the current scaling limit.

## Additional Information

**How to cite this article**: Han, U.-B. and Lee, J.-S. Integration scheme of nanoscale resistive switching memory using bottom-up processes at room temperature for high-density memory applications. *Sci. Rep.* **6**, 28966; doi: 10.1038/srep28966 (2016).

## Supplementary Material

Supplementary Information
