# Supplementary material for: Integration scheme of nanoscale resistive switching memory using bottom-up processes at room temperature for high-density memory applications
Source: Sci Rep. 2016 Jul 1;6:28966. doi: 10.1038/srep28966 (PMC4929478; doi:10.1038/srep28966)
Supplement: Supplementary Information [file srep28966-s1.pdf]

Supplementary Information

Integration scheme of nanoscale resistive switching memory using bottom-up processes at room temperature for high-density memory applications

Un-Bin Han and Jang-Sik Lee*

Department of Materials Science and Engineering, Pohang University of Science and Technology (POSTECH), Pohang 790-784, Korea

*E-mail: jangsik@postech.ac.kr

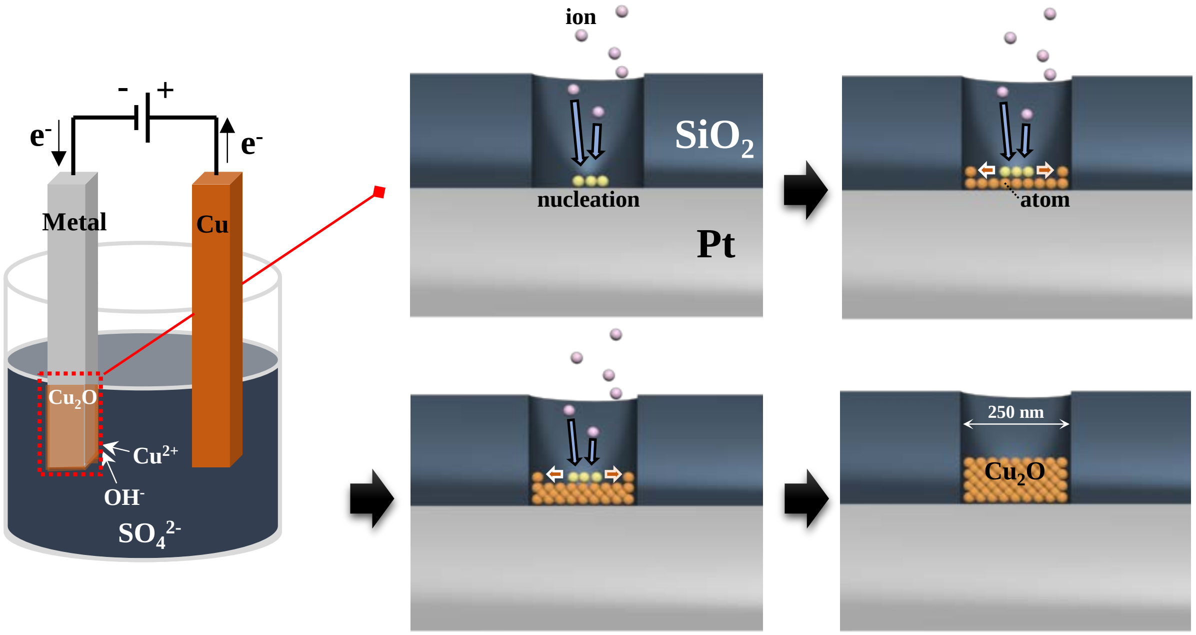

Figure S1. Schematic illustration of bottom-up growth using ECD. Purple, yellow, and orange particles represent ions, adatoms, and stable atoms, respectively. Blue and red arrows represent ion diffusion and surface diffusion.

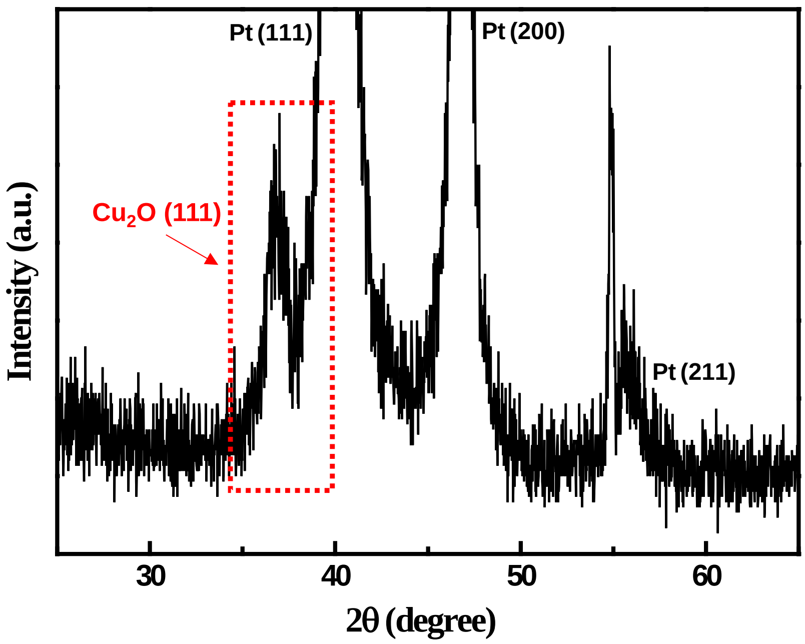

Figure S2. XRD pattern of the Cu_2O film synthesized by electrochemical deposition on Pt-coated substrate.

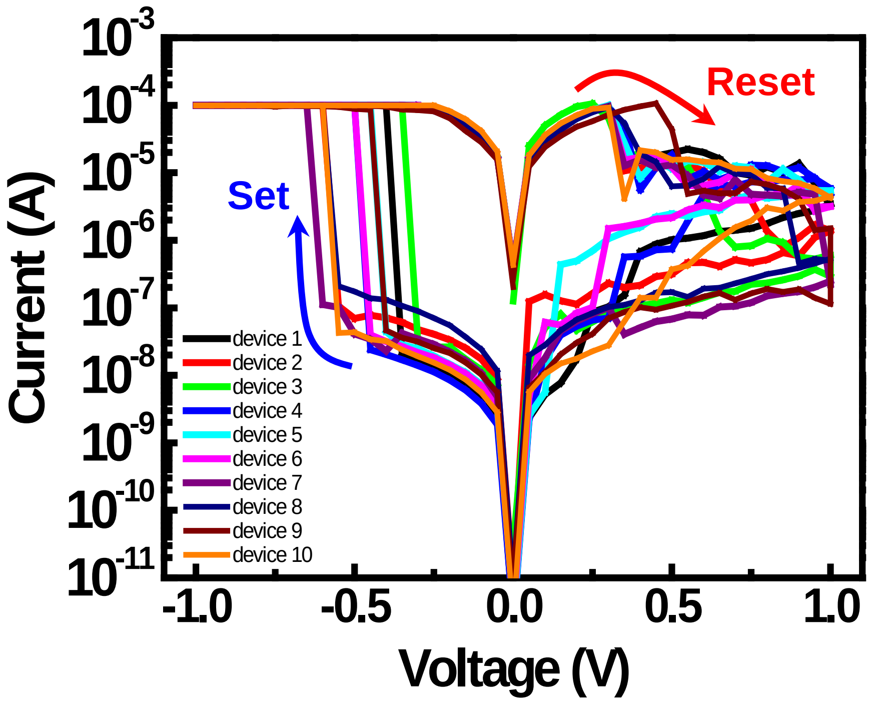

Figure S3. DC I-V curves of the $Pt/Cu_2O/Pt$ devices obtained from various memory cells.

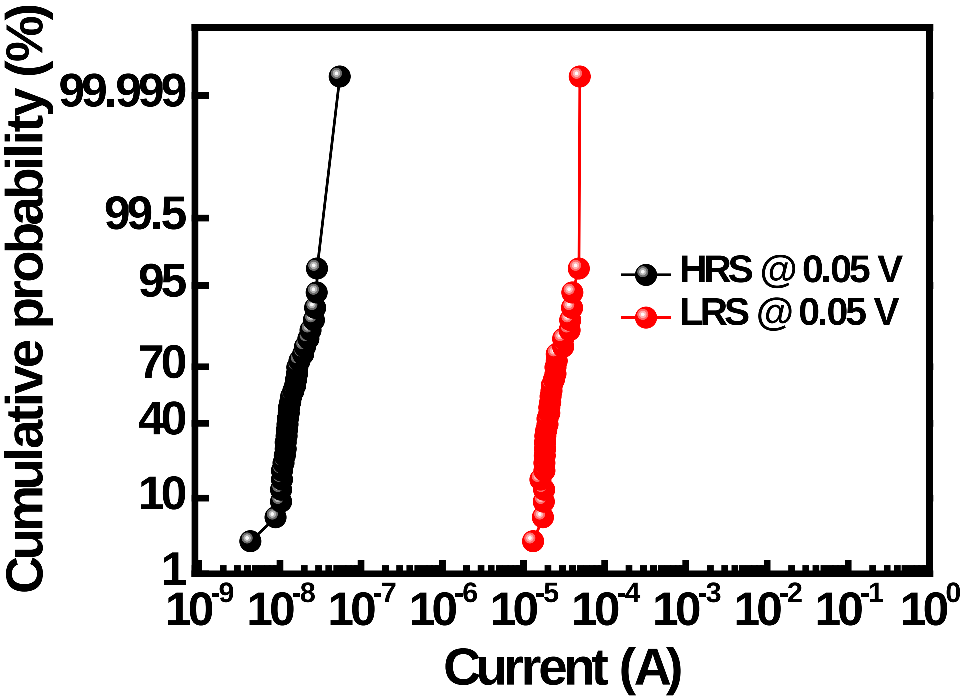

Figure S4. Cumulative probability distribution of Cu_2O-based nanoscale memory devices.
